# Do Père David's Deer Lose Memories of Their Ancestral Predators?

**DOI:** 10.1371/journal.pone.0023623

**Published:** 2011-08-24

**Authors:** Chunwang Li, Xiaobo Yang, Yuhua Ding, Linyuan Zhang, Hongxia Fang, Songhua Tang, Zhigang Jiang

**Affiliations:** 1 Key Laboratory of Animal Ecology and Conservation Biology, Institute of Zoology, Chinese Academy of Sciences, Beijing, China; 2 Graduate University of the Chinese Academy of Sciences, Beijing, China; 3 Dafeng Milu National Nature Reserve, Dafeng Jiangsu, China; 4 Beijing Milu Ecological Research Center, Beijing, China; Australian Wildlife Conservancy, Australia

## Abstract

Whether prey retains antipredator behavior after a long period of predator relaxation is an important question in predator-prey evolution. Père David's deer have been raised in enclosures for more than 1200 years and this isolation provides an opportunity to study whether Père David's deer still respond to the cues of their ancestral predators or to novel predators. We played back the sounds of crows (familiar sound) and domestic dogs (familiar non-predators), of tigers and wolves (ancestral predators), and of lions (potential naïve predator) to Père David's deer in paddocks, and blank sounds to the control group, and videoed the behavior of the deer during the experiment. We also showed life-size photo models of dog, leopard, bear, tiger, wolf, and lion to the deer and video taped their responses after seeing these models. Père David's deer stared at and approached the hidden loudspeaker when they heard the roars of tiger or lion. The deer listened to tiger roars longer, approached to tiger roars more and spent more time staring at the tiger model. The stags were also found to forage less in the trials of tiger roars than that of other sound playbacks. Additionally, it took longer for the deer to restore their normal behavior after they heard tiger roars, which was longer than that after the trial of other sound playbacks. Moreover, the deer were only found to walk away after hearing the sounds of tiger and wolf. Therefore, the tiger was probably the main predator for Père David's deer in ancient time. Our study implies that Père David's deer still retain the memories of the acoustic and visual cues of their ancestral predators in spite of the long term isolation from natural habitat.

## Introduction

Antipredator responses, the later vigilance behaviors such as scan, alert call and flee, evolve to allow animals to minimize their risk of predation [1]–[5]. Stare and approaching to predators is another behavioral response of prey which is taken into account by researchers [6]–[7]. During a long history of coevolution, animals selectively retained ability to respond to the cues of their predators [8]–[11]. Moreover, the oldfield mice, *Peromyscus polionotus*, even uses indirect (microhabitat structure or moonlight) rather than direct cues (urine of native or nonnative predators) to assess risk of predation [12]. Previous studies indicated that the loss of predators may lead to rapid loss of antipredator behavior [13]. However, others suggested that antipredator response may persist for many generations [14], [15]. The divarication for this question may be explained by the Multipredator Hypothesis and the period of isolation from predator.

Multipredator Hypothesis assumes prey respond to extinct predators as long as they had experience with the predators and the prey still have experience with other predators; that is antipredator behavior persists under predation relaxed selection [16]. Although some predators disappear for a long time in the range of a prey, some prey, such as western grey kangaroos *Macropus fuliginosus* [17], and yellow-bellied marmot *Marmota flaviventris* [18], still retained antipredator response to the cues of those predators. However, McPhee (2003) reported that, in oldfield mice *Peromyscus polionotus subgriseus*, individuals from populations that had been kept in captivity for multiple generations sought refuge less often than their wild counterparts [19]. In Tammar wallabies (*Macropus eugenii*), for example, isolation of approximate 130 years from predators resulted in a complete loss of antipredator response [13]. The prey's memory of their predator may relate to the length of period it has been isolated from their predator. However, after 30 generations in captivity, guinea pigs *Cavia aperea* still retained the behavior of their wild counterparts [20].

Père David's deer (*Elaphurus davidianus*), which originally lived in northeastern and east-central China, Korea and Japan, is one of the few large mammals that is extinct in the wild but safely preserved in captivity [21], [22]. Previous paleontological studies indicated that many carnivores, included tiger (*Panthera tigris*), wolf (*Canis lupus*), and bear (*Ursus arctos*), lived with Père David's deer in the same habitat in ancient times [22]–[24]. Père David's deer apparently became extirpated in the wild at least 1200 years ago, since then, this deer has been kept in captivity [21], [22], [24], [25], [26].

We tested the hypothesis that extant Père David's deer retain antipredator responses to the acoustic and visual cues of their ancestral predators. If Père David's deer had already lost the memory of the ancient sympatric predator, they would have acted to the cue of tiger as they do to any naïve predator, such as African lions (*Panthera leo*). To test our hypothesis, we conducted field experiments with sounds playback of ancestral predator, potential predator and non-predator and showing life-size photo models of ancestral or potential predators to Père David's deer. As described in previous studies, the sound playbacks and the photo models were effective in sampling antipredator behavior of deer [9], [27].

## Methods

### Study sites and populations

Our study was carried out in 2008–2009. In this study, we adhered to the ‘Guidelines for the use of animals in research’ published in Animal Behaviour 1991, and also adhered to the Wild Animals Protection Law of the People's Republic of China. All animals in this study were cared under animal research protocol IOZ-2006 approved by the Animal Care Committee of Institute of Zoology, Chinese Academy of Sciences, and cared for in accordance with the principles and permissions approved by Dafeng Père David's Deer Nature Reserve and Beijing Milu Park, respectively.

Dafeng Père David's Deer Nature Reserve (32°59′–33°03′N, 120°47′–120°53′E) and Beijing Milu Park (39°7′N, 116°03′E) of China were used to be two study sites. Père David's deer in our present study were the forth or fifth generation of those deer that were reintroduced from England to these two sites for *ex situ* conservation in 1980s [23], [28]. The fenced area of this reserve was enlarged from original 1000 ha in 1986 (year established) to 2660 ha in 1996. Annual average temperature is 14.1°C, with mean temperature of 0.8°C in January and 27°C in July. Average annual precipitation is about 1,068 mm. There are more than 1,500 deer in the reserve that forms three free ranging populations and a wild population. We carried out our experiment of sound playback on a free ranging population. In this population, there were 108 deer in the fall of 2008, including 50 adult males, 37 adult females, and 21 yearlings and newborns. Beijing Milu Park was chosen as study site for photo model displaying experiment. The park has an area of 60 ha. Annual average temperature is 13.1°C, with mean temperature of −3.4°C in January and 26.4°C in July. Average annual precipitation is about 600 mm. Deer in the study population graze on natural vegetation in summer and autumn with supplementary feeds year round. There were 121 deer in this Park in spring of 2009, including 28 adult males, 52 adult females, 41 two-year old and yearlings.

We videotaped behavioral responses of adult deer (stags and hinds) during the trials. 50 stags and 37 hinds in Dafeng group were repeatedly observed during the sound playback trials in Dafeng. The 28 stags and 52 hinds were observed during the photo model trials in Beijing. All trials were carried out between 8 and 10 a.m., and individuals were sampled only once for each trial. All individuals were distinguished by ear tags. Antler shape and facial characteristics were employed as aids to identify individuals when the ear tags on the deer were unclear. All subjects can receive auditory stimuli because the deer walked and grazed in groups in the enclosures, the live-sized predator photos were displayed to the deer gathered at the feedlot during the feeding time in Beijing Milu Park, while the experimenters were hidden behind a shelter [29], [30].

### Playback experiment

We downloaded the animal sounds for the sound playback trials from the website (http://www.ilovewavs.com/Effects/Animals/Animals.htm), including the common caw of crow (*Corvus corone*), bark of dog, roars of tiger and lion, and howl of wolf. Among those animals, crow and domestic dog live together with Père David's deer in the reserve [31]. Tiger and wolf were ancestral predators of Père David's deer, and lion was a naïve predator that never appeared in the historical range of Père David's deer [24], [25], [32]. The sound of blank (background noise of electric current) was played in the control trial. We randomly arranged the sequence of the animal sounds in playback trials. Each sequence of sound playback trials (or the photo model trials that described in next paragraph) was repeated three more times on three days respectively, and only one of them was randomly chosen and used for the statistical analysis. Duration of each sound playback lasted 1 minute. The interval between sound playback trials was around 30 minutes. To avoid the experimenter influence on the deer, we dressed in camouflage coat, sidled deer, and hid in bushy hassock [9]. When we played back sounds to the deer, the average distance between the sound source and the subjects was 119±11 meters; the acoustic intensity of each sound was 115 decibels at one meter from the loud speaker. We used a digital video camera (Canon XM2, video frame rate is 30 frames per second) to record the behaviors of the deer before, during and after each sound playback trial. Duration of each recording was about 30 minutes.

The videos were replayed on computer in the laboratory and the Focal Sampling Method [33] was used to record the behaviors of each subject. We recorded one individual at a time and collected behaviors that occurred during the entire 30 minutes (10 minutes before sound playback and 20 minutes after sound playback). After we had finished analyzing the record of one deer, then we replayed the video again to record the behavior of another individual. Based on previous studies [5], [6], [7], [30], [34], we recorded antipredator behavior such as stare and approaching (deer stared at and walked towards stimuli source), alarm call, pawing ground for alert, walking-away and flee when they were foraging. We also recorded the duration of behavioral restoration of deer after each sound playback trial.

### Predator photo model experiment

By using a Nikon D100 digital camera and a Cannon iPF9110 color printer, we photographed and created life-size photo models of a domestic dog, leopard (*Panthera pardus*), tiger, lion, bear and wolf. We built a camouflage canvas shelter near the feed lot in the Beijing Milu Park 10 days before our experiment. The photo model was displayed in a random order when the deer were feeding at feed lot. Duration of showing photo model of each predator to the deer lasted 10 minutes. The interval between experiments was around 30 minutes. The distance between the photo model and the deer was approximately 30 meters. We showed a 2*2 m plywood board to the deer as a control trial.

We used a digital video camera (Canon XM2) to record behavior of all deer in three 10-minutes duration before, during and after the image display trials. All videos were replayed on computer in the laboratory and the behaviors of the deer were extracted by using the similar protocol described in the section of *Experiment of Sound Playback*.

### Data analysis

We used SPSS-13 (SPSS, Inc., Chicago, IL, U.S.A.) to test the differences of the frequency of behavioral responses among different trials. When the distribution of behavioral frequencies differed significantly from the normal distribution (one sample Kolmogorov-Smirnov test, *P*<0.05), we then used the Friedman non-parametric 2-way ANOVA to check the differences in the frequency of behavioral response among different trials. The duration of behavioral restoration after sound played back of adults was in accord with the normal distribution (one sample Kolmogorov-Smirnov test, *P*>0.05), the Mauchly's Test of Sphericity showed that the error covariance matrix of the orthonormalized transformed dependent variables was proportional to an identity matrix (*P* = 0.549). Additionally, each trial in our experiment was not independent to other trials. Therefore, we used Repeated Measures of General Linear Model (GLM) to check the difference of those variables among different trials. In this procedure, we calculated the effect size of influencing factors (i.e. the value of partial Eta squared in ANOVA [35], [36], The intraclass correlation coefficient (R) can be defined as the proportion of the total variance accounted for by differences among groups, and were commonly used to represent the common measure of repeatability [37], [38]. We therefore calculated this coefficient to show the level of consistency of individual trajectories. When the difference of behavioral response among different trials was significant, the Multiple Comparison (the Post Hoc test for the parametric analysis and the Wilcoxon test for the nonparametric analysis) between any two trials was done. All data were presented as mean ± standard error unless otherwise specified. The difference at *P*<0.05 was taken as significantly different for all statistical tests.

## Results

### Behavioral changes during sound playbacks

In stags and hinds, frequency of all behaviors except walking way, showed significant differences among six sound play back trials (Friedman Test, df = 5, *P*<0.05, Table 1). The control group grazed more. The lower frequencies of foraging were found in the playbacks of tiger roars, lion roars (only in hinds) and dog barks (only in hinds). Peak frequencies of stare and approaching were found in the trials of playback of roars of tiger and lion. The lowest frequencies of stare and approaching were recorded in the control trial.

**Table 1:** Behavioral changes of Père David's deer during sound playback trials (Occurrences/1 min, 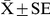).

Behaviors		Trials						χ^2^	*P*
		Control	Caw of crow	Bark of dog	Roar of lion	Roar of tiger	Howl of wolf		
Foraging	Stags	5.36±0.26^a^	2.84±0.36^b^	2.16±0.35^b^	1.62±0.29^b^	1.08±0.28^c^	3.32±0.34^b^	81.91*	0.000
	Hinds	5.67±0.21^a^	3.16±0.41^b^	0.70±0.29^c^	1.00±0.34^c^	0.22±0.17^c^	2.65±0.46^b^	68.08*	0.000
Stare and approaching	Stags	0.48±0.23^c^	2.12±0.32^b^	3.52±0.36^b^	4.38±0.29^a^	4.74±0.29^a^	2.44±0.34^b^	94.42*	0.000
	Hinds	0.29±0.21^c^	1.57±0.37^b^	3.72±0.46^b^	4.67±0.38^a^	5.13±0.31^a^	2.51±0.47^b^	83.26*	0.000
Walking away	Stags	0.00±0.00	0.00±0.00	0.00±0.00	0.00±0.00	0.00±0.00	0.00±0.00	-	-
	Hinds	0.00±0.00	0.00±0.00	0.00±0.00	0.00±0.00	0.00±0.00	0.00±0.00	-	-

*There are significant difference among trials (Friedman Test, df = 5, *P*<0.05). Between any two trials, data with different superscript character (a, b, or c) differed significantly (Wilcoxon Test, *P*<0.05).

### Behavioral changes after sound playbacks

Frequencies of all behaviors in stags showed significant differences in sound playback trials (Friedman Test, df = 5, *P*<0.05, Table 2). Frequency of foraging was higher in control group than those in other groups; whereas, the lower frequency of foraging was found in the trial of tiger roars. The peak frequencies of stare and approaching and walking-away were found in the tiger roars playback trial. The lowest frequency of stare and approaching was found in the control trial.

**Table 2:** Behavioral changes of Père David's deer after sound playback trials (Occurrences/1 min, 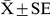).

Behaviors		Trials						χ^2^	*P*
		Control	Caw of crow	Bark of dog	Roar of lion	Roar of tiger	Howl of wolf		
Foraging	Stags	5.36±0.26^a^	3.58±0.39^b^	4.02±0.39^b^	4.30±0.36^b^	2.34±0.37^c^	4.44±0.34^b^	41.25*	0.000
	Hinds	5.68±0.21^a^	2.70±0.47^b^	1.38±0.36^b^	2.48±0.46^b^	0.95±0.34^c^	2.62±0.43^b^	66.99*	0.000
Stare and approaching	Stags	0.48±0.23^c^	1.90±0.36^b^	1.74±0.37^b^	1.24±0.29^b^	3.28±0.39^a^	1.02±0.27^b^	41.82*	0.000
	Hinds	0.30±0.21^c^	0.97±0.34^c^	3.02±0.44^a^	2.32±0.42^a^	2.89±0.46^a^	1.75±0.37^b^	41.23*	0.000
Walking-away	Stags	0.00±0.00^b^	0.00±0.00^b^	0.00±0.00^b^	0.00±0.00^b^	0.16±0.92^a^	0.00±0.00^b^	15.00*	0.010
	Hinds	0.00±0.00	0.00±0.00	0.00±0.00	0.00±0.00	0.00±0.00	0.08±0.06	10.00	0.075

*There are significant difference among trials (Friedman Test, df = 5, *P*<0.05). Between any two trials, data with different superscript character (a, b, or c) differed significantly (Wilcoxon Test, *P*<0.05).

Frequencies of all behaviors in hinds except walking-away after sound playback showed significant differences in six trials (Friedman Test, df = 5, *P*<0.05, Table 2). Foraging in hinds had maximal frequency in the control trial; whereas, the lower frequencies of foraging were found in playbacks of tiger roars. The peak frequency of stare and approaching in stags was found in playback of tiger roars; while, the peak frequency of stare and approaching in hinds were found in playback of tiger roars, lion roars and dog barks. The lowest frequency of stare and approaching was recorded in the control trial. We recorded three times of flee in the trial of tiger roars but non in other trials.

### Alarm call in sound playback trials

**Figure 1:**
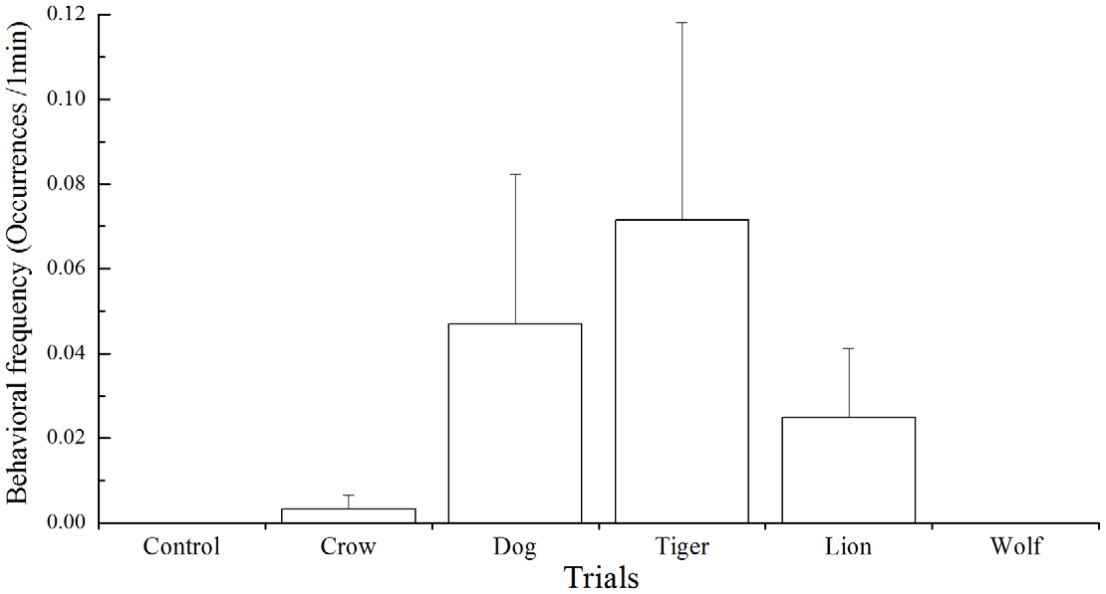
Frequencies of alarm call made by adult Père David's deer in different sound playback trials (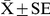).

Frequency of alarm call in adult Père David's deer showed no significant difference among six trials of sound playback (Friedman Test, χ^2^ = 4.68, df = 5, *P* = 0.456. Fig. 1). The peak frequency of alarm call was found in the tiger roars playback trial.

### Durations of behavioral restoration after sound playbacks

**Figure 2:**
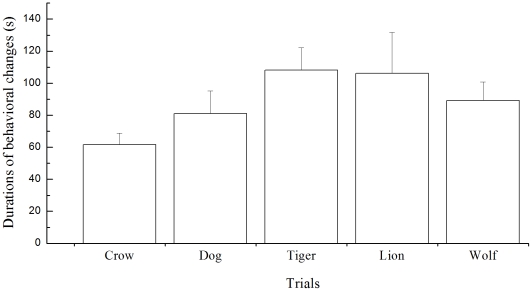
Durations of behavioral restoration in Père David's deer in different trials of sounds played back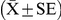. Statistical parameters of the Mauchly's test of sphericity: approximate χ^2^ = 8.048, df = 9, *P* = 0. 549.

There was no significant difference in the durations for the deer to restore their normal behavior in all sounds playback trials (Repeated measures of GLM, F = 2.244, df = 4, *P* = 0.09. Fig. 2). For the effect size of sounds playback trials, partial Eta squared was 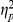 = 0.243, and the measure of repeatability was R = 0.891.

### Behavioral responses to animal photo models

**Figure 3:**
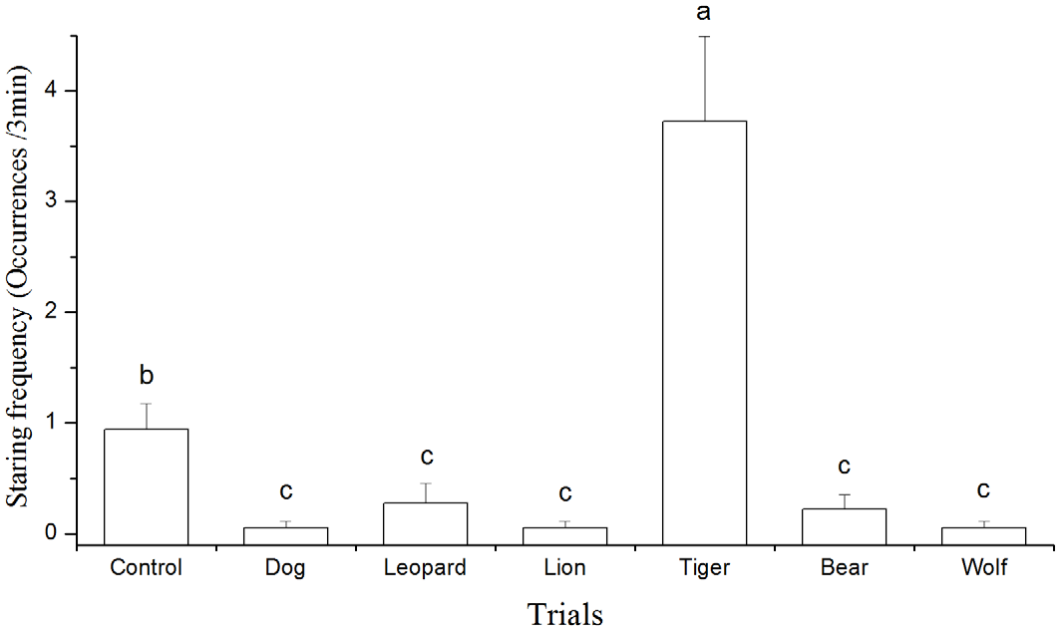
Frequencies of stare at photo models by Père David's deer while feeding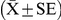. Between any two trials, data with different superscript character (a, b, or c) differed significantly (Wilcoxon Test, *P*<0.05).

Stare behavior in Père David's deer differed significantly among those seven animal photo model displaying trials (Friedman non-parametric 2-way ANOVA, χ^2^ = 50.46, df = 6, *P* = 0.000. Fig. 3). Père David's deer stared more frequently at the tiger model than at the models of other predators and the control. However, we did not observe other antipredator behaviors such as walking-away, flee, alarming call and pawing ground in the experiment.

## Discussion

Père David's deer responded to cues of ancestral or potential novel predators. When they heard the played back sounds, Père David's deer stared at and approached the roars of tigers and lions more than other sound sources. When the sound playback finished, Père David's deer firstly stared and then approached closer to the roars of tigers than other sounds, and before walking further from the source of tiger roars. The change of foraging was another behavioral response that reflected animals' vigilance to predators [39]. Li et al. (2011) found that when the presence of predation risk was high, marmots increased the proportion of time spent vigilant and decreased the time spent foraging [40]. In our present study, Père David's deer stags foraged less in the trials of tiger roars than that of other sound playbacks.

Additionally, it took longer for the deer to restore their normal behavior after they heard tiger roars, which was longer than that after the trial of other sounds. Moreover, Père David's deer were only found to walk away after hearing the sounds of tiger and wolf. Our results also indicated that Père David's deer spent more time to stare at tiger model. That is to say, the roar and the model of tiger were the cues of high predation risk for Père David's deer.

Therefore, Père David's deer still retained the memory of their ancestral predators, such as the tiger and wolf [24], [32], [41]. Based on our study, in contrast to the tiger, Père David's deer did not exhibit strong reactions when they heard or saw the cues of wolves. Presumably, the tiger was the most important predator for Père David's deer in history whereas the wolf was presumably less active in the swamps where Père David deer lived before their extinction in the wild. Our data indicated that Père David's deer showed more vigilance and forged less when they heard the roar of lion (a novel predator for Père David's deer). Salo et al. (2007) suggested that naïve, alien predators had more severe impacts on prey than native predators. Thus, it is important to consider naïve predation risk when release animals to the field of new environment [42].

Alarm calls in adults were recorded, but the occurrences of alarm calls showed no significant difference among different trials of sound playback. Similarly, pig-tailed langur (*Simias concolor*) did not retain specific acoustic knowledge of vocalizations of felid predator but respond to those vocalizations as novel stuffs [43]. It seems that, for contemporary Père David's deer, sound of predators is not so much the predation risk as the novelty. However, another possible reason for this non-significant result was that the responses of each individual within the group were not independent due to our experimental design with repeated-measures. Because the statistic method we used was nonparametric, we did not calculate the repeatability of alarm calls. But in data analysis of the duration of behavioral restoration, the high value of the repeatability indicated that there was high between-individual variation [38]. We supposed that the non-independency, to some extent, resulted in the limits of our study.

As to Multipredator Hypothesis, Stankowich and Coss (2007) found that mule deer (*Odocoileus hemionus*) exhibited stronger antipredator response to their current predator than to jaguar, a locally extinct predator, and suggested that prolonged relaxed selection has led to the loss of recognition of prey's historical predators [9]. In addition, we found that the variation of behavioral response of Père David's deer showed more changeful in the trials of sound playback than in photo model experiment. We assumed that Père David's deer respond to the acoustic and visual cues in different ways.

Père David's deer have been isolated from all predators for more than 1200 years, but the deer still responded to the sounds and images of their ancestral predators. Evidence indicated that antipredator behavior of island rodents in response to cues of fox predators was not likely to be rapidly lost by removing fox (*Urocyon littoralis*) [15]. However, dissimilar result was found in moose (*Alces alces*) in Yellow Stone National Park, North America [44]. The moose were unfamiliar with dangerous predators after as short as 50 to 130 years of predation relaxation, apparently, they lost memories of their ancestral predators, but they could recover the antipredator behavior to reduce predation within a single generation [44]. By this token, behavior of prey evolved together with their predators due to predator–prey arms races [45], [46]. Experience dependent behavior may be lost after the first generation of isolation, but more “hard-wired” antipredator behavior may persist for thousands years after the isolation of nature predators [47], [48].
